# Suppressing H_2_ evolution by using a hydrogel for reversible Na storage in Na_3_V_2_(PO_4_)_3_

**DOI:** 10.1039/c9ra08402a

**Published:** 2020-01-02

**Authors:** Xianying Fan, Xiaoyu Gao, Xuan Zhang, Guijia Cui, Huichao Lu, Zhixin Xu, Jun Yang

**Affiliations:** Shanghai Electrochemical Energy Devices Research Center, Department of Chemical Engineering, Shanghai Jiao Tong University Shanghai 200240 China yangj723@sjtu.edu.cn

## Abstract

We report a low-cost hydrogel electrolyte by adding 3 wt% poly(acrylate sodium) (PAAS) into 1 M Na_2_SO_4_ aqueous electrolyte, which achieves a widened electrochemical stability window (ESW) of 2.45 V on stainless steel current collector from 2.12 V in 1 M Na_2_SO_4_ aqueous electrolytes (AE). Moreover, the H_2_ evolution potential reaches −1.75 V *vs.* Ag/AgCl on titanium current collector. The results reveal that the polymer network structure of PAAS has the ability to interact with water molecules and thus the hydrogen evolution reaction can be limited effectively, which broadens the ESW of aqueous electrolyte and allows the reversible Na-ion intercalation/deintercalation of Na_3_V_2_(PO_4_)_3_ as an anode material in aqueous electrolyte reported for the first time.

## Abstract 2

Hydrogen evolution suppression of hydrogel electrolyte with 3 wt% poly(acrylate sodium) (PAAS) ensures reversible Na-ion storage of Na_3_V_2_(PO_4_)_3_ as negative electrode.

## Graphical abstract

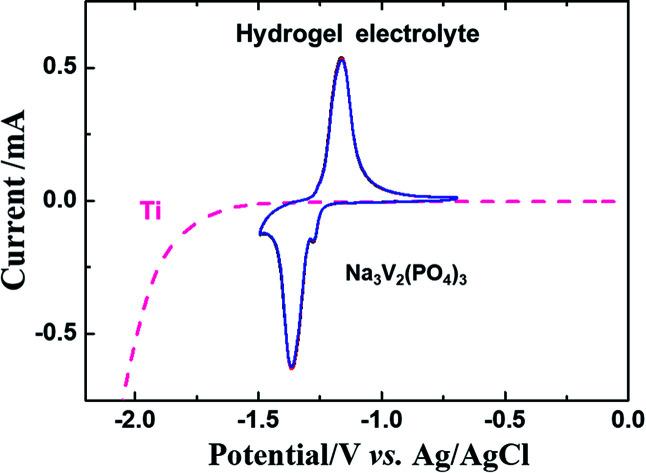

## Introduction

Sodium-ion batteries have attracted much attention in the field of energy storage systems due to the rich abundance and low cost of sodium. Nonetheless, conventional sodium-ion batteries are based on toxic and flammable organic electrolytes which may cause security issues in practical applications.^1–5^ Aqueous electrolytes for sodium-ion batteries have the advantages of non-flammability, high ionic conductivity, and low cost. However, the thermodynamic ESW of water as an electrolyte solvent is restricted to barely 1.23 V arising from the H_2_ and O_2_ evolution, and thus the output voltage and energy density of cells are limited.^5–14^ Recently, Wang *et al.*^8^ and Battaglia *et al.*^9^ have made remarkable progress on high concentration (9.26 M) sodium trifluoromethanesulfonate (NaCF_3_SO_3_) and ultrahigh (up to 37 M) concentration sodium bis(fluorosulfonyl)imide (NaFSI) in water by forming “water-in-salt” electrolyte (WiSE), which has been the most effective approach to bind “free” water for extending stable electrochemical windows to more than 2.5 V. But the exorbitant cost due to the massive use of organic sodium salts and temperature-dependent solubility of salts hinder the practical application of the WiSE strategy. Therefore, a simple and economical strategy to suppress the electrochemical decomposition of water for aqueous sodium batteries is of great importance.

Besides, it is also meaningful to select an electrode material that is compatible with the electrolytes to achieve Na-ion storage. Nowadays, the often used anode materials, such as activated carbon (2–2.6 V *vs.* Na/Na^+^),^15^ NaTi_2_(PO_4_)_3_ (2.1 V *vs.* Na/Na^+^),^16^ vanadium oxide Na_2_V_6_O_16_·*n*H_2_O (2.5 V *vs.* Na/Na^+^),^12^ or organic anodes polyimide (2.2–2.4 V *vs.* Na/Na^+^),^17,18^ possess relatively high electrode potentials in aqueous sodium-ion batteries, which leads to low cell voltage output. Na_3_V_2_(PO_4_)_3_ with a NASICON structure has been intensively investigated for sodium-ion batteries as a cathode.^19–24^ In fact, Na_3_V_2_(PO_4_)_3_ has the characteristics of dual potential plateaus at 3.4 V *vs.* Na^+^/Na and 1.6 V *vs.* Na^+^/Na, and the lower one has been utilized for anode use in Na_3_V_2_(PO_4_)_3_ symmetrical full cells with non-aqueous electrolytes that show good cycle stability and high rate capability.^23,24^ Provided Na_3_V_2_(PO_4_)_3_ could be also used anode in aqueous electrolytes, a higher cell voltage can be expected. However, since its Na^+^-intercalation potential (1.6 V *vs.* Na/Na^+^) is much lower than hydrogen evolution potential (2.3 V *vs.* Na/Na^+^),^5^ finding a special aqueous electrolyte that fits Na_3_V_2_(PO_4_)_3_ anode is important.

Herein, we introduce a new aqueous electrolyte based on the combination of inexpensive Na_2_SO_4_ salt and hydrogel poly(acrylate sodium) (PAAS). The interaction among salt, PAAS and water molecules suppresses hydrogen evolution reaction effectively, which broadens the ESW of aqueous electrolyte and allows the reversible Na-ion intercalation/deintercalation of Na_3_V_2_(PO_4_)_3_ as an anode in aqueous electrolyte.

## Results and discussion

**Fig. 1:**
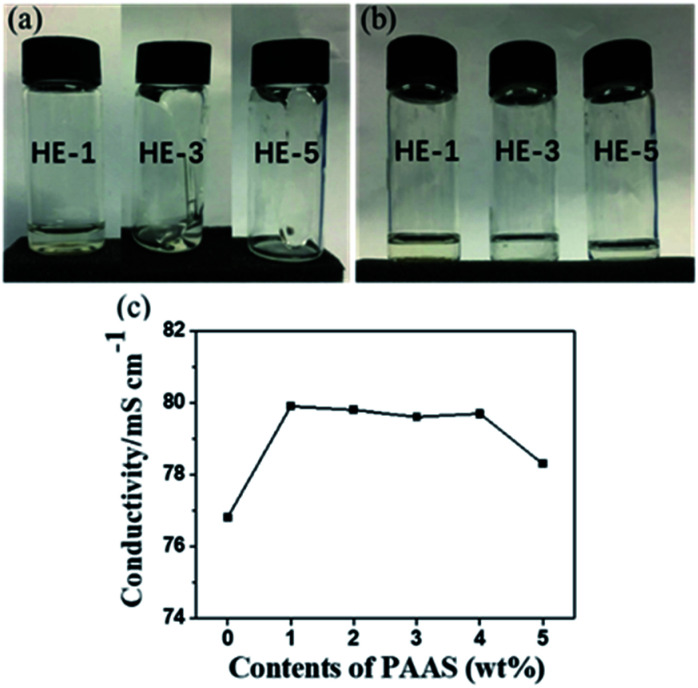
Physical states and conductivities of the electrolytes. The appearances of HEs after flipping the vials for 2 s (a) and for 60 s (b). The conductivities of AE and HEs (c).

Hydrogel electrolytes (HEs) of various concentrations were prepared by mixing 1 M Na_2_SO_4_ aqueous electrolytes with an appropriate amount of PAAS (1, 3, 5 wt% respectively, which are noted as HE-1, HE-3, HE-5, *M*_w_ = 300w–700w, Aladdin), and then by intense stirring at room temperature until dissolved. As shown in Fig. 1a and b, the states of the electrolytes changes from liquid to gel after the addition of PAAS, and the viscosity of the weakly acidic HEs (pH = 5.5–7) increases with higher PAAS content. The conductivity of HEs at room temperature is about 78–80 mS cm^−1^ compared to 76 mS cm^−1^ for AE (Fig. 1c). The higher conductivity with PAAS might be mainly related to the increased Na^+^ content from PAAS. The declined conductivity for 5 wt% PAAS could be explained by the rising viscosity. It is noteworthy that the conductivity of the aqueous gel polymer electrolytes is much higher than those of the organic sodium-ion electrolytes (6–12 mS cm^−1^) and most of “water-in-salt” electrolytes (∼10 mS cm^−1^).^5,8,9^

**Fig. 2:**
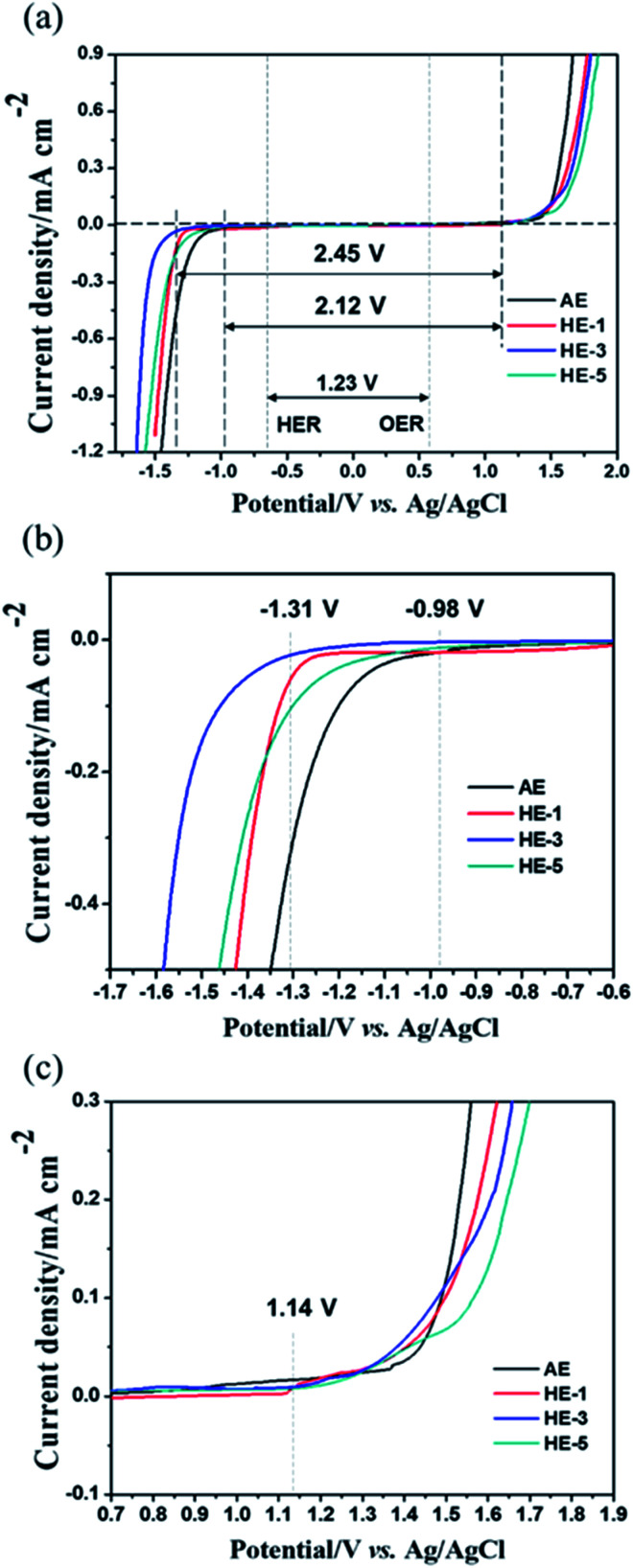
Potentiodynamic scan of electrolytes on a stainless-steel working electrode. The electrochemical stability windows of AE and HEs, scan rate: 10 mV s^−1^. (a) The enlarged regions near anodic and cathodic extremes of the electrochemical stability windows on stainless steel current collector (b) and (c).

Fig. 2 shows the ESW of HEs obtained by potential dynamic polarization using a stainless steel (SS) working electrode in a three-electrode cell. The addition of various amounts of PAAS to the AE results in different degrees of ESW broadening and the content of 3 wt% PAAS (HE-3) exerts the strongest effect. Using HE-3, there is a sharp over-potential increase of about 330 mV for H_2_ evolution reaction (HER). That is, the initial HER potential shifts from −0.98 V for AE to −1.31 V *vs.* Ag/AgCl for HE-3, and the total ESW is as wide as 2.45 V compared to 2.12 V in AE. It may be ascribed to the strong water absorption effect of PAAS which causes self-ionizing H^+^ of water molecules to interact with –COO^−^, resulting in a decrease in the mobility of H^+^ in HEs and negative shift of the hydrogen evolution potential.

**Fig. 3:**
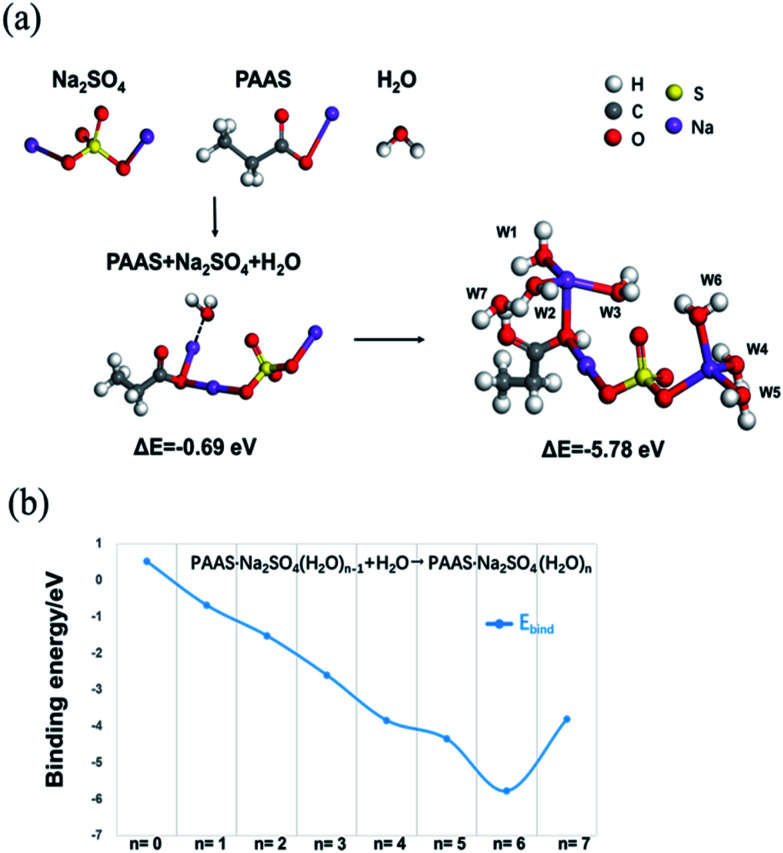
Relative binding energies at 298 K of PAAS, Na_2_SO_4_ and H_2_O from DFT calculations using Dmol3 model (a). Binding energies (*E*_bind_) for the Na^+^ cation to approach multiple water molecules (*n* = 0–7) as calculated by DFT calculations with Dmol3 model (b).

To better explain the function of PAAS in 1 M Na_2_SO_4_ aqueous electrolyte, we have used DMol3 method based on density functional theory (DFT)^25,26^ to calculate the binding energy, and determine the possible combined forms of Na^+^ and oxygen atoms with water molecules in the cluster. The results of the binding energy changes among PAAS, Na_2_SO_4_ and water molecules are provided in Fig. 3a. The binding energy between the above three is −0.69 eV (<0), which indicates that PAAS and Na_2_SO_4_ have some interaction with water molecules in HEs. This may be due to the similar attraction effect between –COO^−^ or SO_4_^2−^ to Na^+^ that makes PAAS and Na_2_SO_4_ preferentially form a more structurally stable molecular cluster to restrain the water molecule tightly. Na^+^ ions from both PAAS and the Na_2_SO_4_ have solvation with water molecules and the remaining unsolvated water molecules act with the carboxylate in a manner to form hydrogen bonds (Fig. 3a and b, W1–W7 and Table 1). Moreover, the widened ESW may be also related to the interaction of ion-pair charges between –COOH groups, which are formed by self-ionizing H^+^ and –COO^−^ groups. The charge interaction between ions expands the polymer network and causes a large osmotic pressure, which enhances the interaction between water molecules and oxygen atoms and also raises salt tolerance ability of PAAS^27^ at the same time. Since the part of H^+^ exists in the form of a –COOH group, the reduction resistance of the water molecules is slightly enhanced. These interactions jointly lead to the improvement in cathodic stability observed in HEs as shown in Fig. 2b.

**Table 1:** Relative energies of PAAS·Na_2_SO_4_(H_2_O)_*n*_ (*n* = 1–7) clusters as calculated by DFT calculations with Dmol3 model

Molecules	*E* [Ha]	Δ*E* [Ha]	Δ*E* [eV]
C_2_H_5_COONa	−427.206724	—	—
Na_2_SO_4_	−1018.656642	—	—
H_2_O	−75.861179	—	—
C_2_H_5_-COONa–Na_2_SO_4_·H_2_O (W1)	−1521.749738	−0.025193	−0.69
C_2_H_5_-COONa–Na_2_SO_4_·2H_2_O (W2)	−1597.641585	−0.055861	−1.52
C_2_H_5_-COONa–Na_2_SO_4_·3H_2_O (W3)	−1673.542468	−0.095565	−2.60
C_2_H_5_-COONa–Na_2_SO_4_·4H_2_O (W4)	−1749.449321	−0.141239	−3.84
C_2_H_5_-COONa–Na_2_SO_4_·5H_2_O (W5)	−1825.329216	−0.159955	−4.35
C_2_H_5_-COONa–Na_2_SO_4_·6H_2_O (W6)	−1901.242812	−0.212372	−5.78
C_2_H_5_-COONa–Na_2_SO_4_·7H_2_O (W7)	−1977.031559	−0.13994	−3.81

**Fig. 4:**
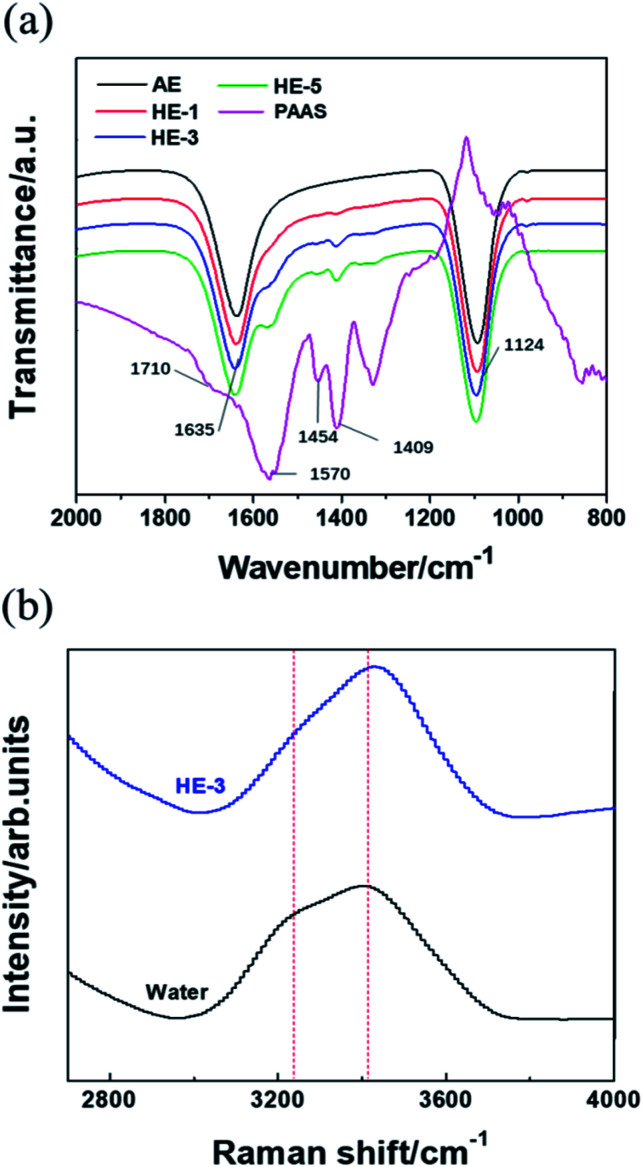
FTIR spectra of PAAS, HEs and AE (a). Raman spectra of HE-3 and pure water (b).

Fig. 4a shows the Fourier transform infrared (FTIR) analysis of PAAS, Na_2_SO_4_ and water molecules. The strong peak at 1124 cm^−1^ shown in AE and HEs can be attributed to the antisymmetric stretching vibration of SO_4_^2−^. For PAAS, the peak at 1454 cm^−1^ indicates the angle vibration absorption of –CH_2_, while the peaks at 1570 and 1409 cm^−1^ correspond to the antisymmetric and symmetric stretching vibration absorption peaks of –COO^−^, respectively. These three characteristic peaks become more pronounced with the increase of PAAS content. The stretching vibration absorption peak of C=O (1710 cm^−1^) disappears when PAAS is added into aqueous solutions, indicating that the combination of the water molecules with carboxylate oxygen may weaken the C=O stretching vibration absorption peak. Besides, the H–O–H bending vibration is red-shifted from 1630 cm^−1^ to 1635 cm^−1^ when the concentration of PAAS increased from 0% (AE) to 5 wt% (HE-5), which is likely due to an increase of viscosity.

It can be observed in Fig. 4b that HE-3 shows a narrower peak compared to the broad Raman band from 3000 cm^−1^ to 3700 cm^−1^ of pure water. In addition, the intensity of the peak at 3200 cm^−1^ corresponding to free water is lower and the peak of coordinated water shifts to 3550 cm^−1^ from 3410 cm^−1^. Hence, it can be inferred that HE-3 can achieve better electrochemical stability due to the combination of a fraction of water molecules.

**Fig. 5:**
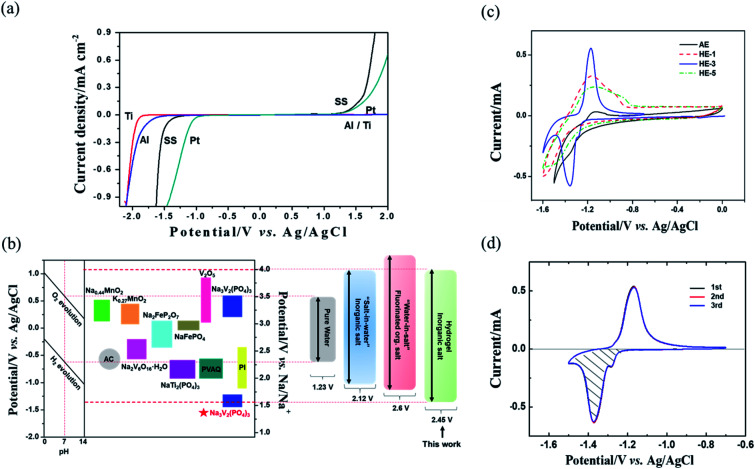
The electrochemical stability windows of HE-3 on different working electrodes, scan rate: 10 mV s^−1^ (a). The intercalation potential of some electrode materials that could possibly be employed for aqueous sodium-ion storages and comparison of aqueous sodium-ion storages end-of-charge voltages with various salts (b). CV of Na_3_V_2_(PO_4_)_3_ working electrode (coated on titanium) in a three-electrode system containing HEs and AE for the first cycle at scanning rate of 5 mV s^−1^ (c). CV of Na_3_V_2_(PO_4_)_3_ working electrode (coated on titanium) in a three-electrodes cell containing HE-3 for the first three cycles from −0.7 V to −1.5 V *vs.* Ag/AgCl at scanning rate of 5 mV s^−1^ (d).

The material type of the anode current collector has an apparent influence on the HER over-potential. We further measured the ESW of HE-3 by potentiodynamic scanning on different metal electrodes, including stainless steel (SS), Al, Ti and Pt (Fig. 5a). Al is a typical current collector and can suppress the HER potential to −1.55 V *vs.* Ag/AgCl compared with Pt (−1.0 V) or SS (−1.31 V). Furthermore, the HER potential for Ti electrode can reach −1.75 V (*vs.* Ag/AgCl) in HE-3. The HER over-potential of the anode current collectors in HE-3 follows the sequence: Ti > Al > SS > Pt.

**Fig. 6:**
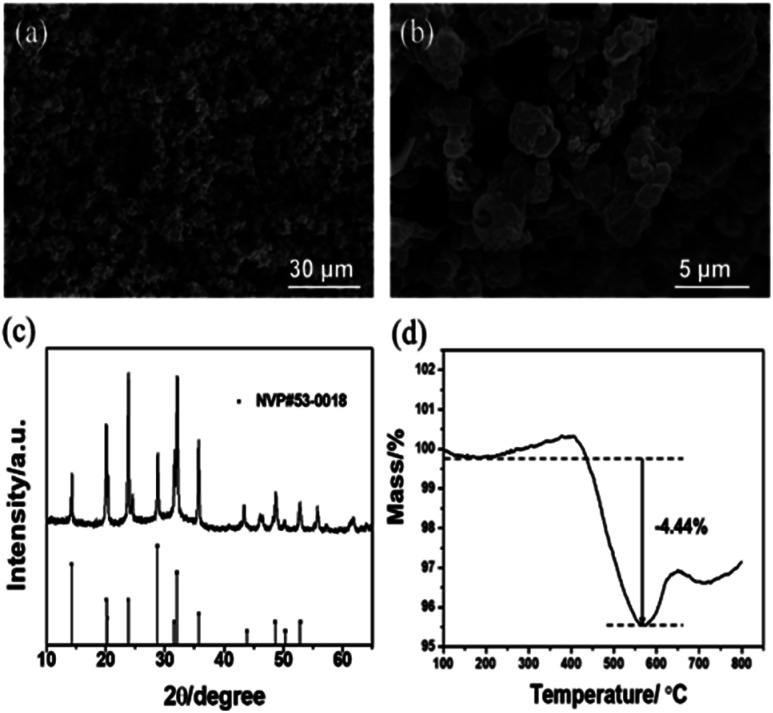
SEM images (a and b), XRD pattern (c) and TG curve (d) of the as-prepared Na_3_V_2_(PO_4_)_3_.

The electrode materials that have been reported in aqueous electrolyte systems are shown in Fig. 5b. The widened ESW of HE-3 provides more options for electrode materials available for reversible sodium-ion storage. We have selected NASICON structured Na_3_V_2_(PO_4_)_3_ material for a potential Na^+^ intercalation electrode with titanium current collector. The synthesized Na_3_V_2_(PO_4_)_3_ has irregular particle shapes in the sizes mostly below 5 μm (Fig. 6a and b). Its XRD pattern in Fig. 6c accords with the standard one well. TG result indicates that the Na_3_V_2_(PO_4_)_3_ material contains 4.44% carbon (Fig. 6d), and the weight increases after 550 °C could be attributed to the oxidation of V^3+^ in Na_3_V_2_(PO_4_)_3_.^21,22^Fig. 5c shows cyclic voltammograms of Na_3_V_2_(PO_4_)_3_ electrode in the three-electrode system containing HEs and AE at a scan rate of 5 mV s^−1^. As evidenced by the results in Fig. 5c, the intercalation of sodium-ion in Na_3_V_2_(PO_4_)_3_ electrode is accompanied by hydrogen evolution reaction in AE, HE-1 and HE-5. Due to the strong competing reduction, no obvious Na^+^ deintercalation peak can be observed in AE. On the other hand, although the Na^+^ intercalation and hydrogen evolution potentials cannot be distinguished in HE-1 and HE-5, the corresponding Na^+^ deintercalation peaks are still distinct, confirming the PAAS effect in Fig. 2. By contrast, the potential of Na^+^ intercalation in HE-3 is well distinguishable from that of hydrogen evolution despite their small difference. The symmetric redox peaks at −1.36 V and −1.18 V *vs.* Ag/AgCl (*i.e.* 1.56 V and 1.74 V *vs.* Na/Na^+^), accorded with the reported results,^21–24^ demonstrate the reversible sodium-ion intercalation/deintercalation of Na_3_V_2_(PO_4_)_3_ in HE-3. This result proves that HE-3 effectively broadens the ESW of aqueous electrolyte and allows the reversible Na-ion intercalation/deintercalation of Na_3_V_2_(PO_4_)_3_ as an anode in aqueous electrolyte.

The electrochemical cycle reversibility of Na_3_V_2_(PO_4_)_3_ has been further examined by multiple CV test from −0.7 V to −1.5 V (*vs.* Ag/AgCl). As shown in Fig. 5d, the reduction peaks and oxidation peaks appear at approximately −1.38 V and −1.19 V (*vs.* Ag/AgCl) respectively for the initial three cycles. These three cycles are highly coincident, reflecting the good Na^+^-intercalation/deintercalation reversibility of Na_3_V_2_(PO_4_)_3_ electrode in HE-3. In addition, the Na^+^ storage capacity of Na_3_V_2_(PO_4_)_3_ can be estimated from the reversible cyclic voltammetry (CV) curves using the following eqn (1).^28^1
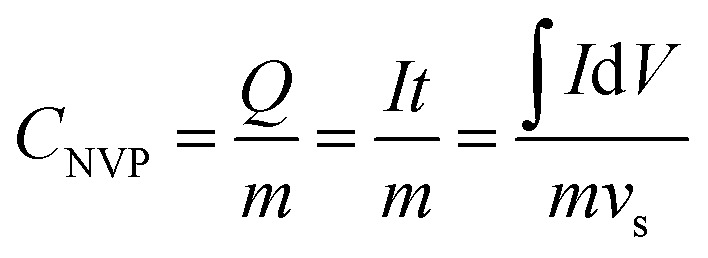
*m* = 0.467 mg, *v*_s_ = 5 mV s^−1^, ∫*I*d*V* = 309.793 mA Vwhere *C*_NVP_ and *m* are the specific capacity and mass of Na_3_V_2_(PO_4_)_3_ material, *Q* refers to the quantity of electric charge, which is equal to the product of current (*I*) and time (*t*), that is, the ratio of the shaded area (∫*I*d*V*) under the CV curve to the scan rate (*v*_s_).

The specific capacity of Na_3_V_2_(PO_4_)_3_ electrode was calculated from CV curves (Fig. 5d) and eqn (1). The specific capacity of Na_3_V_2_(PO_4_)_3_ electrode is about 37.2 mA h g^−1^ during the reduction process. Although this capacity is lower than the previously reported capacity of Na_3_V_2_(PO_4_)_3_ in non-aqueous electrolyte (67 mA h g^−1^),^23^ it should be mentioned that the dynamic factors, such as the particle size and scanning rate, could influence the available capacity. The reversible electrode reaction provides the possibility for the research on aqueous Na_3_V_2_(PO_4_)_3_-based cells and other aqueous cell systems using an anode that has a lower potential than the frequently used NaTi_2_(PO_4_)_3_.

## Conclusions

We have investigated the PAAS hydrogel electrolyte containing 1 M Na_2_SO_4_ for potential use in aqueous Na-ion batteries. It is found that the PAAS content has obvious influence on the hydrogen evolution potential. Adding 3 wt% PAAS to 1 M Na_2_SO_4_ aqueous electrolyte effectively inhibits the hydrogen evolution. The ESW of aqueous gel electrolyte is broadened to an extent of 330 mV from 2.12 V (in AE) to 2.45 V (in HE-3) on stainless steel current collector, and particularly the hydrogen evolution potential is shifted to −1.75 V *vs.* Ag/AgCl (*i.e.* 1.17 V *vs.* Na/Na^+^) on titanium current collector. DFT calculation and spectroscopic analysis confirm the interaction between water molecules and PAAS, which may decrease the water activation. Finally, through CV test and cathodic area calculation we first prove the feasibility of Na_3_V_2_(PO_4_)_3_ anode for reversible Na storage in this electrolyte with the stable capacity of *ca.* 37.2 mA h g^−1^.

## Experimental

### Electrolytes preparation

Aqueous electrolyte (AE, 1 M anhydrous sodium sulfate (Na_2_SO_4_, Sinopharm Chemical Reagent Co., Ltd) in deionized water (HPLC grade)) was prepared according to molality (mol-salt in kg-solvent). The hydrogel electrolytes (HEs) were prepared by mixing 1 M Na_2_SO_4_ solution with different ratios (1 wt%, 3 wt%, 5 wt%, named HE-1, HE-3, HE-5, respectively) of poly(acrylate sodium) (PAAS, *M*_w_ = 300w–700w, Aladdin), and then followed by intense stirring under room temperature until dissolved.

### Material synthesis and electrode preparation

The Na_3_V_2_(PO_4_)_3_ powder was synthesized by spray drying method as reported, in which a given amount of NH_4_VO_3_ (99.9%, Macklin), NH_4_H_2_PO_4_ (99%, Aladdin), Na_2_CO_3_ (99%, Energy Chemical) and glucose (as the carbon precursor and the reductive agent) were dissolved in deionized water and stirred at 80 °C until blue clear solution formed. The blue solution was spray-dried to obtain the precursor by a spraying dryer (BUCHI Mini Spray Dryer B-290), where the inlet and outlet temperatures were set at 200 °C and 105 °C, respectively, the feed pump speed was 8% and the gas speed was 5000 mL min^−1^. Then, the obtained powder was pre-calcined in a tube furnace at 350 °C for 5 h and calcined at 750 °C for 12 h in N_2_ atmosphere. The Na_3_V_2_(PO_4_)_3_ electrodes were prepared as followed: Na_3_V_2_(PO_4_)_3_ material, Super P and polyvinylidene fluoride (PVDF) were mixed with a weight ratio of 8 : 1 : 1 in *N*-methyl-2-pyrrolidone (NMP), which was spin-coated on a titanium grid, and was dried at 80 °C for 8 h under vacuum condition.

### Material characterizations

FTIR spectra were measured on a PerkinElmer Spectrum 100. Raman spectra were collected with a DXR Raman Microscope using a 532 nm diode-pumped solid-state laser between 4000 and 2700 cm^−1^, with all the samples sealed in a glass test tube. The scanning electron microscopy (SEM) images of Na_3_V_2_(PO_4_)_3_ powder were obtained on Hitachi-X650 microscope (20 kV) scanning electron microscopy. The X-ray diffraction (XRD) patterns were recorded on a scan rate of 2°·min^−1^ from 10° to 65° by using Rigaku diffractometer (*D*_max_-2200) with Cu–K radiation. The carbon content in Na_3_V_2_(PO_4_)_3_ powder was analyzed using a Thermo Gravimetric Analyzer (TGA/Pyris 1 TGA).

### Electrochemical measurements

Ionic conductivity was measured with Rex conductivity meter DDS-307 at room temperature. The electrochemical stability windows (ESW) of the electrolytes were examined by cyclic voltammetry (CV) using CHI650C electrochemical workstation and the glassy three-electrode system with Ag/AgCl as reference electrode (2.92 V *vs.* Na/Na^+^), stainless steel (SS 304), aluminum (Al), titanium (Ti), platinum (Pt) and prepared Na_3_V_2_(PO_4_)_3_ as working electrodes, and graphite as counter electrode. The experiments were conducted at scanning rate of 10 or 5 mV s^−1^ in a given potential range at 25 °C.

## Conflicts of interest

There are no conflicts to declare.

## Supplementary Material
